# Learning from the challenges of Ebola Virus Disease contact tracers in Sierra Leone, February, 2015

**DOI:** 10.11694/pamj.supp.2015.22.1.6537

**Published:** 2015-10-11

**Authors:** Olayinka Stephen Ilesanmi

**Affiliations:** 1 Department of Community Health, Federal Medical Centre, Owo, Ondo State, Nigeria; 2 Africa Union Support to Ebola Outbreak in West Africa, Tonkolili District, Sierra Leone

**Keywords:** Ebola, outbreak, Sierra Leone, contact tracers, epidemic

## Abstract

**Introduction:**

Sierra Leone was in the process of strengthening tracing of Ebola Virus Disease (EVD) contact with training of contact tracers, continuous mentoring and monitoring, supervision and continuous support. This was through various national and international organizations. This study aimed at identifying the challenges of contact tracers with a view of improving contact tracing activities in Tonkolili District, Sierra Leone.

**Methods:**

In-depth interview was conducted among contact tracers who were actively involved in contact tracing within the 4 weeks preceding the interview. In-depth interview guide was used to interview the contact tracers. Questions were asked about the state of EVD outbreak, challenges of contact tracing, ways to improving contact tracers activities and ways to ensure community participation and follow up action.

**Results:**

A total of 12 Contact tracers were interviewed. Most of the contact tracers saw the lifting of ban by the Government on movement as a delay to stopping the outbreak. Some of them were being threatened by their communities and insulted. Some communities with EVD cases felt it was no longer in Sierra Leone and that the contact tracers were the ones infecting the people with Ebola. More than 80% of the participants indicated that retraining of contact tracers and re-orientation of community members would help in putting a stop to the outbreak.

**Conclusion:**

All participants indicated interest in improving their activities and performance. They suggested that more social mobilization is needed to ensure the cooperation of their communities.

## Introduction

The ongoing Ebola Virus Disease (EVD) outbreak in West Africa is large and difficult to contain [1]. This is with respect to the number of countries and people affected. Stopping the outbreak require concerted efforts. The strategies identified in stopping the EVD include patient identification and isolation, contact tracing and improving community understanding with safe patient and body transport systems, safe burial and environmental decontamination [2]. Contact tracing aims to identify and monitor contacts of known Ebola cases in order to isolate and investigate them for Ebola as early as possible if they become ill. This should prevent any further transmission and allow those who are ill to get care early. Seeking treatment early is likely to improve the disease outcome. Contact tracing is a form of surveillance it is not limited to EVD only but it is one of the most important pillars in communicable disease control and prevention [3]. Contact tracing teams identify the contacts. Aside identification they follow them up daily in their households for 21 days by asking them about symptoms. Contacts are defined by the modes of transmission of Ebola. Most contacts will be household members but there will also be some contacts in the community. Risk of contacts varies. Contact who cleansed a dead body positive to EVD is at high risk, but others attending the funeral are also contacts, particularly if they touch the body.

Contact tracing can be beneficial if the correct information is released to the contact tracer. If the tracer does his or her work diligently a lot can be achieved. However, some factors have been identified to affect contact tracing. The issue of stigma and discrimination could hamper good contact tracing. Though psychologically for the contacts it may not be easy on them but it involves everyone, the community and their understanding. Community understanding will enable the members to cooperate with the contact tracers. Fear of discrimination made contacts not to give correct information. Some community did not agree with the offer of having contact tracers with them. In Sierra Leone some community members were identified to have thrown stones at health workers participating in investigating the outbreak [4]. Effective contact tracing in Africa setting is highly dependent on community engagement and involvement to succeed. This is as a result of the respect Africans have for traditional institutions and leadership especially in the rural areas. This should be leveraged on by encouraging traditional leaders to be actively involved in encouraging members of their communities to cooperate and support not only the contact tracers but the entire control efforts. In addition to what is known, there is a need to identify the challenges of the contact tracers to offer solution and ensure quality contact tracing activities. This study aimed at identifying the challenges contact tracers encounter with a view to improving contact tracing activities in Tonkolili District, Sierra Leone.

## Methods

### Participants

These are contact tracers that have participated in the tracing of contact in the last 4 weeks before the interview was conducted. The interview were limited to active contact tracers. Overall, 16 contact tracers were in the inclusion criteria. However, saturation of ideas was achieved after interviewing the twelfth contact tracer. In-depth responses about the contact tracer's experiences and challenges were collected. Few verbatim quotations were included. All the participants were 22 years and older.

### Procedure

Invitation was giving to the participants during a meeting with their mentor and monitor. Participation of contact tracers was voluntary. All participants were advised that they could withdraw from the study at any time. The response rate was 100%. No one refused to participate and non-withdrew their participation from this study. Questions were asked about the state of EVD outbreak, challenges, ways to improving contact tracers activities, ways to ensure community participation and follow up action. Interview questions were unstructured and designed to promote open-ended responses. Interview was between 15-20 minutes. Interview response were written by the interviewer and another observer. The study took place at Tonkolili District of Sierra Leone at a time when at least 6 households were quarantined. Using contact tracers attached to communities that were not having contacts would not be able to bring out the current challenges the contact tracers were faced with. The study participants were informed that their response will help in improving their activities on the field. The results were validated by comparing them with the challenges identified in other parts of the outbreak response programme aside contact tracing activities. This data is worth analyzing due to the differences in the social context in which the contact tracers work. They have worked at their various communities for a minimum of 6 weeks. The researcher's potential influence was minimized by ensuring that the data was collected by a neutral researcher who is neither monitoring nor mentoring the contact tracers. The data was collected in February, 2015 after the presidential announcement that restriction on movements was lifted in Sierra Leone. This was a time when a steady state was observed in the outbreak trend.

### Ethical considerations

This study was based on data collected during surveillance and response activities for EVD in Sierra Leone. All the information collected on individual patients and contact tracers were anonymous to improve surveillance activities.

## Results

A total of 12 Contact tracers were interviewed. The analysis of interviews revealed five main themes: state of the EVD outbreak, challenges, assessment of ways to improve contact tracers activities, assessment of ways to ensure community participation and follow up action.

### State of EVD outbreak

Every contact tracer spoke about the occurrence of EVD in the district. They reacted to the lifting of ban on movements in different ways. Most of them saw the lifting of ban by the Government as a source of delay to end the outbreak. One of the main causes of the cases seen in unsuspected areas are the customs, habits, and beliefs of the affected communities. This includes burial practices and patronage of traditional healers. These factors are important to be considered in health education for the scourge of EVD to end. Some of these cultural factors, hallowed by centuries of practice, have stood in the way of implementing health programmes that will bring an end to the outbreak. It was notable that where a change of behaviour was necessary, the resistance of the people was maximum in accepting new programmes. “My people are just difficult”. “It seems nothing is working because of their fixed mind set about health issues” said one of the respondents. One contact tracer reported that understanding the people and their social belief can be the starting point of any intervention. Another factor identified to have caused an increase in the magnitude of the outbreak is low level of education of the community members. Education of the individuals and family will help in improving positive health habits. However, such must be tailored towards the individuals and families and built on their existing knowledge. Other factors that may be beneficial to the communities must also be considered since health behaviours are not governed by a single set of attitudes. A person may consider frequent hand washing not to prevent the transmission of EVD but other diseases.

### Assessment of challenges

All the contact tracers saw the lifting of ban by Government on movement as a delay to stopping the outbreak. Half of the contact tracers interviewed said that “In the last few weeks, the new cases in the district came from outside the district”. The community thought Ebola Virus Disease was over and it was no longer in Sierra Leone. Two contact tracers said they were accused of infecting the community with Ebola. Some community members have a misconception that some of the contact tracers have come to cause outbreak. Another contact tracer added that some community members feel that Ebola does not kill but the activities that follow. Some communities blame the contact tracers when a case was picked. They see the contact tracers as exposing the community. This is not limited to only the community members some traditional rulers do not understand how Ebola is spread. They felt once a household is quarantined nobody can get EVD in the community aside the members of quarantined households. The support needed by the contact tracers from the community for surveillance activities was not usually made available, before questions were asked they were always eager to say no. However, wet cases were seen in some of the quarantine homes occasionally. Some villages lacked mobile telephone network this hindered communications with supervisors. Going to another village to make phone calls delayed communication flow. Some of them were being threatened by their communities and insulted.

### Ways to improve contact tracers activities

Re-training of contact tracers was identified as a way to improve their activities. This would help them to proffer solutions to identified challenges. Contact tracers who are performing well should also be rewarded. Likewise those who have had several quarantine homes to trace should be given extra compensation. More community support was also encouraged.

### Ways to ensure community participation

Denial rate was high in the communities more than nine months to the commencement of the outbreak. One respondent narrated that “I was told by the chief in my community that Ebola may not be the cause of death of some of the community members since people died in non-quarantine homes also”. Social mobilization activities was suggested by the respondents as a means of increasing the knowledge of the community members about Ebola Virus Disease.

### Follow up action

Most of the interviewed contact tracers indicated that more passion should be applied to their activities in all the villages. Only two of them felt they have done all that is within their capacity. Four others suggested that Ward councilors should organize meetings at community level to present them (contact tracers) to the chiefs and community members. This was seen as a way of improving community acceptance. Maintenance of cross boundary check points until the outbreak is over was suggested to the ward counsellors to ensure zero case through the support of the paramount chiefs. More than 80% of the participants indicated that retraining of contact tracers and re-orientation of community members would help in ending the outbreak.

## Discussion

Prior research has shown that for an effective and result-oriented contact tracing, it is pertinent to have a public awareness campaign to educate the affected community on the importance of the exercise. This will enable the community to see the need to give authentic information since they are directly or indirectly affected. Education and prevention will help fight stigma with respect to Ebola Virus Disease as it has done for HIV AIDs [5]. The decline of the outbreak of EVD most likely resulted from changes in the community′s behaviours, such as altering traditional burial practices so that people could avoid catching the virus from dead patients. Getting the message out into the community and getting people to change their behaviour is critical if we are to bring the current outbreak under control. Measures such as isolating and treating patients, effective contact tracing and well-coordinated surveillance activities, and health education of the community are all part of effective response [6].

Direct sensitization such as performing door-to-door campaigns and organizing community meetings to discuss the disease were shown to have led to positive changes in health related behavior [7]. Both rural and urban population, have their peculiar beliefs and practices concerning health and disease. However, social mobilisation and community sensitization should be utilized as a key component because all stakeholders should be involved to enable them to pool resources and optimize the management of EVD cases and contacts [8]. The role community based intervention plays cannot be over emphasized [9]. Behaviour change is a process [10]. Habit-formation generates recommendations for simple and sustainable behaviour change [11]. This calls for intervention by the health sector itself to take the initiative towards strong workable partnerships with other sectors locally and internationally. This would not only produce the possible benefits of inter-sectoral synergy, symbiosis, peer review, and efficiency but could also enhance the health status of the people [12].

Perhaps the biggest challenge was improving public awareness of the disease, and trust and confidence in the medical response. Good communication, transparency, and community engagement will be central to success [4]. Community chiefs should be involved in ensuring the support of their community members. The members of the community are the only one who can have easy access to the community. Community awareness needs to continue until the communities are seen playing their expected roles. The most successful public health programs and initiatives are based on an understanding of health behaviours and the context of its occurrence. Therefore, relevant theories of behaviour change and the ability to use them skillfully should be the premise on which interventions to improve health behaviour can best be designed. Information about factors like customs, cultural mores, habits, beliefs, and superstition are important for any health changing behavior to be accepted [13]. This factors need to be worked on for the community to accept change.

## Conclusion

Contact tracing is critical in controlling the EVD outbreak. It is more of a community action towards epidemic outbreak investigation, surveillance, education, awareness training and participation. Efforts of contact tracers will be futile if the community is not participating adequately. All the contact tracers interviewed were interested in improving their activities and performance. They suggested that more social mobilization is needed to ensure the co-operation of their communities.
